# Genomic Treasure Troves: Complete Genome Sequencing of Herbarium and Insect Museum Specimens

**DOI:** 10.1371/journal.pone.0069189

**Published:** 2013-07-29

**Authors:** Martijn Staats, Roy H. J. Erkens, Bart van de Vossenberg, Jan J. Wieringa, Ken Kraaijeveld, Benjamin Stielow, József Geml, James E. Richardson, Freek T. Bakker

**Affiliations:** 1 Biosystematics Group, Wageningen University, Wageningen, The Netherlands; 2 Maastricht Science Program, Maastricht University, Maastricht, The Netherlands; 3 Ecology and Biodiversity Group, Department of Biology, Utrecht University, Utrecht, The Netherlands; 4 Dutch National Plant Protection Organization, National Reference Centre, Wageningen, The Netherlands; 5 Netherlands Centre for Biodiversity Naturalis (section NHN), Herbarium Vadense (WAG), Wageningen University, Wageningen, The Netherlands; 6 Department of Human Genetics/Leiden Genome Technology Center, Leiden University Medical Center, Leiden, The Netherlands; 7 Centraalbureau voor Schimmelcultures Fungal Biodiversity Centre (CBS-KNAW), Utrecht, The Netherlands; 8 Naturalis Biodiversity Center, Section National Herbarium of the Netherlands, Leiden, The Netherlands; 9 Royal Botanic Garden Edinburgh, Inverleith Row, Edinburgh, United Kingdom; 10 Laboratorio de Botánica y Sistemática, Universidad de Los Andes, Apartado Aéreo 4976, Bogotá, Colombia; University of Florence, Italy

## Abstract

Unlocking the vast genomic diversity stored in natural history collections would create unprecedented opportunities for genome-scale evolutionary, phylogenetic, domestication and population genomic studies. Many researchers have been discouraged from using historical specimens in molecular studies because of both generally limited success of DNA extraction and the challenges associated with PCR-amplifying highly degraded DNA. In today's next-generation sequencing (NGS) world, opportunities and prospects for historical DNA have changed dramatically, as most NGS methods are actually designed for taking short fragmented DNA molecules as templates. Here we show that using a standard multiplex and paired-end Illumina sequencing approach, genome-scale sequence data can be generated reliably from dry-preserved plant, fungal and insect specimens collected up to 115 years ago, and with minimal destructive sampling. Using a reference-based assembly approach, we were able to produce the entire nuclear genome of a 43-year-old *Arabidopsis thaliana* (Brassicaceae) herbarium specimen with high and uniform sequence coverage. Nuclear genome sequences of three fungal specimens of 22–82 years of age (*Agaricus bisporus*, *Laccaria bicolor*, *Pleurotus ostreatus*) were generated with 81.4–97.9% exome coverage. Complete organellar genome sequences were assembled for all specimens. Using *de novo* assembly we retrieved between 16.2–71.0% of coding sequence regions, and hence remain somewhat cautious about prospects for *de novo* genome assembly from historical specimens. Non-target sequence contaminations were observed in 2 of our insect museum specimens. We anticipate that future museum genomics projects will perhaps not generate entire genome sequences in all cases (our specimens contained relatively small and low-complexity genomes), but at least generating vital comparative genomic data for testing (phylo)genetic, demographic and genetic hypotheses, that become increasingly more horizontal. Furthermore, NGS of historical DNA enables recovering crucial genetic information from old type specimens that to date have remained mostly unutilized and, thus, opens up a new frontier for taxonomic research as well.

## Introduction

As genomic studies are becoming more ‘horizontal’ by comparing genome data from different species, including close relatives of model organisms, the need for well-described and data-based tissue collections will increase. Natural history collections around the world contain an immense number of expert-verified specimens that can contribute invaluable insights to the geographical distribution, phenotypic variation and taxonomy of virtually all known plant, fungal and insect species. The use of such collections is particularly relevant for species that are becoming extinct or increasingly rare (or rather invasive). Natural history collections have played a crucial role at the forefront of biological sciences, and with taxonomic records dating back to the 17^th^ century they have proven invaluable, for instance, for research on biodiversity [1], [2], biological invasions [3] and climate-induced changes in ecology and phenology [4], [5].

Historical DNA sequences have proven extremely informative especially from rare or now extinct species and populations [6]–[8], where markers used were typically short sequences of plastid or mitochondrial-encoded genes for plants and insects respectively [9], [10], or nuclear ribosomal DNA sequences for fungi [11]. Low-copy nuclear genomic sequences, however, have always remained significantly more difficult to obtain from historical DNA. Their acquisition is highly desirable, as they will allow historical specimens to be included in genome-scale evolutionary, domestication and population genomic analyses [12]–[14]. Yet, many researchers have been discouraged from using historical specimens because of both generally limited success of DNA extraction and the challenges associated with PCR-amplifying highly degraded DNA. Due to the outstanding diversity of secondary compounds, including polyphenolics and polysaccharides that can covalently bind to DNA or co-precipitate with DNA, and which are known to inhibit PCR even in non-degraded DNA samples, DNA can be notoriously difficult to extract from plant herbarium tissues. Particular leaf types and textures such as those from succulents (e.g., Crassulaceae, Aloeaceae, Cactaceae), hard- and fibrous-leaved species (e.g., Aquifoliaceae), carnivorous plants, and taxa with resin or sap (e.g., Apocynaceae, Pinaceae, Sapotaceae) can similarly hinder DNA extraction. In addition, historic sample preparation methods can significantly affect DNA recovery success [15]. For decades, a common practice for field preparation, especially in the tropics, was alcohol drying, also known as the Schweinfurth method, to prevent specimens from mould damage. Unfortunately, use of alcohol drying as a temporary fixative is known to have destructive effects on DNA [15]. For insects a commonly-used method is killing with ethyl acetate or formalin-based collecting methods which are known to impede DNA recovery [16]. Considerable effort has been spent on optimizing DNA extraction protocols and, in general, fragments shorter than 300 bp can now be extracted from a broad range of historical specimens [15], [17], [18].

In today's next-generation sequencing (NGS) world, opportunities and prospects for historical DNA have changed dramatically, as most NGS approaches do not rely on large, intact DNA templates but are actually designed for taking short fragmented molecules (100–400 bp) as templates. DNA isolated from historical specimens provides precisely that: the process of specimen preparation which may include exposure to heat (plants/fungi) or killing using ethyl acetate or formalin (insects), is known to cause considerable genome fragmentation by occurrence of extensive double-stranded breaks, and to be independent of specimen age [16], [19], [20]. While the application of NGS technologies to ancient DNA from paleontological and archaeological records has been firmly established [21], [22], its application to historical museum specimens is rare and so far limited to mammals [23]–[25], snails [26] and plants [27], [28].

We set out to investigate the feasibility of obtaining genome-scale sequences using the Illumina HiSeq 2000 platform from a wide-range of historical plant, fungal and insect museum specimens. Both reference-based and *de novo* sequence assembly methods were implemented to test reliability of the assembled sequences. Where possible we compared reads from historical and fresh tissues of the same species to test for elevated sequencing error rates in historical specimens. We selected ‘typical’ museum specimens in order to keep as close as possible to the reality of museum specimens and their preservation histories. We found that complete organellar and nuclear genomes can reliably be generated from low quantities' of historical DNA using NGS.

## Results

### DNA extraction and Illumina sequencing

We obtained sufficient DNA quantities from all plant and fungal specimens to be visually detectable on agarose gel (Figure S1). Total DNA yields extracted from herbarium specimens ranged between 2400 and 45000 ng (Table 1) and the DNA was typically highly degraded with DNA fragment sizes mostly below 1kb (Figure S1). For the plant herbarium specimens, sequencing and quality trimming of low quality nucleotides resulted in data sets containing between 36,926,748 (3.21 giga base pairs, Gbp) and 93,810,738 (8.72 Gbp) reads (Table 2), with read lengths between 87 and 93 nucleotides. Quality trimmed datasets of fresh plant tissue contained comparable numbers of reads and read lengths to those generated for plant herbarium specimens. Quality trimmed data sets of fungal herbarium specimens contained between 23,852,078 (1.93 Gbp) and 50,890,906 (3.87 Gbp) reads (Table 2). The read lengths were between 69 and 89 nucleotides.

**Table 1:** Specimen information, tissue type sampled, DNA yield and DDBJ/EMBL/Genbank accession. See further specimen information in table S2.

Species, type of material	Sample/Collection date	Tissue type sampled (Total DNA yield in ng)	DDBJ/EMBL/Genbank study accession
**Plant:**			
*Arabidopsis thaliana*, herbarium	21 April 1969	Leaf (2400)	ERP001797
*Arabidopsis thaliana*, fresh tissue	July 2010	Leaf (9890)	ERP001798
*Liriodendron tulipifera*, herbarium	28 June 1897	Leaf (3500)	ERP001799
*Liriodendron tulipifera*, fresh tissue	8 July 2010	Leaf (9405)	ERP001800
*Laburnum anagyroides*, herbarium	17 May 1946	Leaf (30000)	ERP001801
*Laburnum anagyroides*, fresh tissue	8 July 2010	Leaf (15000)	ERP001802
**Fungus:**			
*Agaricus bisporus*, herbarium	16 November 1990	Basidiome (15000)	ERP001803
*Pleurotus ostreatus*, herbarium	4 October 1931	Basidiome (8000)	ERP001804
*Laccaria bicolor*, herbarium	7 October 1989	Basidiome (45000)	ERP001805
**Insect:**			
*Aedes albopictus*, archived	December 1999	Complete specimen (650)	-
*Anoplophora glabripennis*, fresh	December 2010	Part of larva stadium (9100)	-
*Anoplophora glabripennis*, archived	July 1992	One rear leg (1500)	ERP001808
*Ceratitis capitata*, fresh	December 2010	Two legs (750), and thorax/head (1000)	ERP001807
*Ceratitis capitata*, archived	April 1995	Three legs (800), and thorax/head (1200)	ERP001806

NASC  =  The European Arabidopsis Stock Centre, Nottingham, UK.

**Table 2:** Alignments of reads generated for archival plant, fungal and insect specimens, and fresh control tissues. All percentages of reads are relative to the number of trimmed reads.

Specimen, type of material	Trimmed reads	Mapped reads (%)		Uniquely mapped reads (%)^1^		Read depth^3^		% Genome coverage^4^		Reference genome	
**Plant herbarium specimen**										Genome	Source
*Arabidopsis thaliana,* herbarium	36,926,748	22,021,533	(59.6)	16,345,196	(44.3)	12.1	(12.8)	94.0	(98.4)	*A. thaliana* nuclear	GCF_000001735.3
*Arabidopsis thaliana*, herbarium	36,926,748	3,771,157	(10.2)	293,443	(0.79)	167.2	(167.2)	100		*A. thaliana* chloroplast	NC_000932
*Laburnum anagyroides*, herbarium	93,810,738	4,169,008	(4.4)	241,721	(0.26)	174.4	(174.7)	99.9		*L. anagyroides* chloroplast	fresh tissue, this study^2^
*Liriodendron tulipifera*, herbarium	54,746,358	2,592,231	(4.7)	311,437	(0.57)	171.4	(171.5)	99.9		*L. tulipifera* chloroplast	NC_008326
**Plant fresh tissue**											
*Arabidopsis thaliana*, fresh tissue	30,898,216	19,780,655	(64.0)	16,344,621	(52.9)	12.1	(12.1)	99.8	(100)	*A. thaliana* nuclear	GCF_000001735.3
*Arabidopsis thaliana*, fresh tissue	30,898,216	3,185,803	(10.3)	307,989	(0.99)	175.4	(175.4)	100		*A. thaliana* chloroplast	NC_000932
*Laburnum anagyroides*, fresh tissue	44,672,406	2,467,061	(5.52)	244,192	(0.55)	176.2	(176.4)	99.9		*L. anagyroides* chloroplast	fresh tissue, this study^2^
*Liriodendron tulipifera*, fresh tissue	52,065,984	8,307,499	(15.9)	316,717	(0.61)	174.3	(174.3)	100		*L. tulipifera* chloroplast	NC_008326
**Fungal herbarium specimen**											
*Agaricus bisporus*, herbarium	23,852,078	13,330,723	(55.9)	10,525,133	(44.1)	28.7	(30.0)	95.4	(97.9)	*A. bisporus* nuclear	H97 v2.0, MycoCosm
*Agaricus bisporus*, herbarium	23,852,078	1,808,428	(7.6)	310,765	(1.30)	156.3	(156.3)	99.9		*A. bisporus* mitochondrion	H97 v2.0, MycoCosm
*Laccaria bicolor*, herbarium	49,124,456	22,591,856	(45.9)	14,969,141	(30.5)	20.7	(29.1)	71.2	(81.4)	*L. bicolor* nuclear	v2.0, MycoCosm
*Laccaria bicolor*, herbarium	49,124,456	1,020,156	(2.1)	81,921	(0.17)	166.1	(166.5)	99.9		*L. bicolor* mitochondrion	herbarium, this study^2^
*Pleurotus ostreatus*, herbarium	50,890,906	23,594,103	(46.4)	15,909,901	(31.3)	35.8	(45.6)	78.4	(88.8)	*P. ostreatus* nuclear	PC15 v2.0, MycoCosm
*Pleurotus ostreatus*, herbarium	50,890,906	238,898	(0.47)	81,226	(0.16)	127.3	(127.4)	99.9		*P. ostreatus* mitochondrion	herbarium, this study^2^
**Archived insect tissue**											
*Anoplophora glabripennis*, archived	49,813,018	29,789	(0.06)	8,994	(0.02)	37.6	(41.4)	90.9		*A. glabripennis* mitochondrion	NC_008221
*Aedes albopictus*, archived	29,343,030	657	(0.002)	594	(0.002)	2.3	(3.5)	63.5		*A. albopictus* mitochondrion	NC_006817
*Ceratitis capitata*, archived (legs)	29,864,834	114,771	(0.38)	24,577	(0.08)	135.4	(135.4)	100		*C. capitata* mitochondrion	NC_000857
*Ceratitis capitata*, archived (head/thorax)	25,896,990	211,743	(0.82)	26,527	(0.10)	146.1	(146.1)	100		*C. capitata* mitochondrion	NC_000857
**Insect fresh tissue**											
*Anoplophora glabripennis*, fresh	6,487,061	31,223	(0.48)	12,563	(0.19)	47.8	(49.8)	95.9		*A. glabripennis* mitochondrion	NC_008221
*Ceratitis capitata*, fresh (legs)	41,781,720	80,323	(0.19)	26,059	(0.06)	143.6	(143.8)	99.9		*C. capitata* mitochondrion	NC_000857
*Ceratitis capitata*, fresh (head/thorax)	32,447,206	118,065	(0.36)	29,045	(0.09)	159.9	(159.9)	100		*C. capitata* mitochondrion	NC_000857

1 The number of uniquely mapped reads after filtering for PCR duplicates.

2 Reference sequence was generated using *de novo* assembly.

3 Average read depth for covered positions, i.e. regions with non-zero coverage only, is given in brackets.

4 Percentage coverage of exonic regions is given in brackets.

For insects, we performed an initial pilot study to assess whether different parts (a single leg or complete thorax and head) of adult specimens of *Ceratitis capitata* would yield sufficient amounts of DNA for sequencing. DNA concentrations, as measured using a NanoDrop 1000 spectrophotometer, extracted from single legs of *C. capitata* were generally low (∼1 ng µl^−1^; not shown) and indeed no or little DNA was visible on agarose gel, hence the integrity of the DNA could not be checked. Based on prior experience with degraded DNA samples at the LGTC, we considered extracts with a minimum DNA yield of ∼600 ng to be suitable for Illumina sample preparation. Therefore, DNA extracts from multiple (two or three) individual legs of the same *C. capitata* specimen were mixed prior to Illumina sample preparation (Table 1). DNA extracted from a single leg of *Anoplophora glabripennis* yielded 1,500 ng DNA. For *Aedes albopictus*, however, the DNA yield for multiple pooled legs was below ∼200 ng and, therefore, DNA was extracted from the entire specimen, which yielded 650 ng DNA (Table 1). Quality-trimmed datasets of archived insect specimens contained between 25,896,990 (2.28 Gbp) and 49,813,018 (3.17 Gbp) reads (Table 2), with read lengths between 55 and 88 nucleotides. Sequence qualities of the reverse read libraries of *A. albopictus* and *A. glabripennis* quickly dropped towards the ends of the Illumina reads (not shown), resulting in relatively short quality-trimmed reads (55 nt). Quality-trimmed datasets of fresh insect tissues contained comparable numbers of reads and read lengths, except for *A. glabripennis* of which the entire reverse Illumina dataset was below the set quality limit. Therefore, only the quality-trimmed forward read dataset generated for fresh tissue of *A. glabripennis* was used.

### Alignment of plant specimen reads

For our 43-year-old *Arabidopsis thaliana* herbarium specimen, reads were aligned using genome assembly TAIR10 (GCF_000001735.3; Chromosomes 1 to 5) as a reference. A total of 22,021,533 reads (59.6% of 36,926,748) mapped to TAIR10 of which 16,345,196 (44.3%) were kept after removing PCR duplicates (Table 2). Genome coverage was 112,003,524 nt (94.0%) and the average read depth was 12.1× (12.8× for covered regions only). Genome coverage and read depth coverage were even across chromosomes (Table S1). To assess the coverage of exonic regions, we used the TAIR10 defined gene features (ftp://ftp.arabidopsis.org/home/tair/Genes/TAIR10_genome_release/TAIR10_gff3/TAIR10_GFF3_genes.gff). We found that 98.4% of the exome was covered, and exonic regions had higher coverage compared to other genomic regions (not shown).

We also used the *A. thaliana* chloroplast genome NC_000932 as reference. A total of 293,443 (0.79%) reads were uniquely-mapped and these covered the entire chloroplast genome. The average read depth was 167.2×.

No chloroplast reference genome is publically available for *Laburnum anagyroides*. We therefore performed *de novo* assembly using reads generated for fresh tissue of *L. anagyroides* and selected scaffolds that mapped to a *Glycine max* chloroplast reference genome [NC_007942] (see methods and Table 3). The final *L. anagyroides* chloroplast reference assembly consisted of 3 scaffolds with a total length of 128,899 nt. Less than 1% of reads mapped uniquely to the *L. anagyroides* and *Liriodendron tulipifera* (NC_008236) chloroplast reference genomes (Table 2). The average read depths for *L. anagyroides* and *L. tulipifera* were 174.4× and 171.4×, respectively, and genome coverage was 99.9% for both chloroplast reference genomes. Similar genome coverages and average read depths were found for DNA sequenced from fresh tissues (Table 2).

**Table 3:** *De novo* assemblies of reads generated for archival plant, fungal and insect specimens, and fresh control tissues.

Specimen, type of material	Velvet settings^1^	Assembly size (Mb)	N50 (nt)	Contigs	Alignable contigs^2^	% Genome coverage^3^	Reference genome
**Plant herbarium specimen**							
*Arabidopsis thaliana*, herbarium	27*	67.1	1,107	65,388	64,024	55.5 (16.2)	*A. thaliana* nuclear, GCF_000001735.3
*Arabidopsis thaliana*, herbarium	47, 50, 150*	0.19	15,211	34	8	98.4	*A. thaliana* chloroplast, NC_000932
*Labunum anagyroides*, herbarium	47, 50, 2000	0.82	3,614	280	3	81.2	*G. max* chloroplast, NC007942
*Liriodendron tulipifera*, herbarium	39, 150*	0.18	22,290	34	6	100	*L. tulipifera* chloroplast, NC_008326
**Plant fresh tissue**							
*Arabidopsis thaliana*, fresh tissue	27*	78.0	1,154	73,838	65,612	60.9 (19.8)	*A. thaliana* nuclear, GCF_000001735.3
*Arabidopsis thaliana*, fresh tissue	47, 50, 150*	0.14	9,114	28	18	97.2	*A. thaliana* chloroplast, NC_000932
*Laburnum anagyroides*, fresh tissue	47, 50, 2000	1.49	12,726	223	3	81.4	*G. max* chloroplast, NC007942
*Liriodendron tulipifera*, fresh tissue	39, 150*	0.21	8,296	43	15	99.2	*L. tulipifera* chloroplast, NC_008326
**Fungal herbarium specimen**							
*Agaricus bisporus*, herbarium	41, 5, 50	27.7	20,217	1,820	1,720	81.1 (71.0)	*A. bisporus* H97 v2.0 nuclear, MycoCosm
*Agaricus bisporus*, herbarium	41, 50, 2000	0.24	41,776	43	2	99.9	*A. bisporus* H97 v2.0 mitochondrion, MycoCosm
*Laccaria bicolor*, herbarium	41, 5, 50	38.8	19,276	2,559	2,495	62.0 (34.4)	*L. bicolor* v2.0 nuclear, MycoCosm
*Laccaria bicolor*, herbarium	51, 50, 1000	1.55	6,809	384	5	24.5	*C. cinerea* okayama7#130 mitochondrion, Broad
*Pleurotus ostreatus*, herbarium	41, 5, 100	34.3	85,861	858	725	78.5 (56.4)	*P. ostreatus* PC15 v2.0 nuclear, MycoCosm
*Pleurotus ostreatus*, herbarium	41, 50, 300	2.24	9,705	405	9	90.2	*P. ostreatus* mitochondrion, EF204913
**Archived insect specimen**							
*Aedes albopictus*, archived^4^	xx	xx	Xx	xx	xx	xx	
*Anoplophora glabripennis*, archived	51, 20*	0.15	2,022	77	0	0	*A. glabripennis* mitochondrion, NC_008221
*Ceratitis capitata*, archived (leg)	39, 150*	0.04	5,249	12	1	96.8	*C. capitata* mitochondrion, NC_000857
*Ceratitis capitata*, archived (head/thorax)	39, 150*	0.03	3,282	11	2	96.8	*C. capitata* mitochondrion, NC_000857
**Insect fresh tissue**							
*Anoplophora glabripennis*, fresh	51, 20*	0.43	3,650	106	5	90.3	*A. glabripennis* mitochondrion, NC_008221
*Ceratitis capitata*, fresh (leg)	39, 150*	0.05	3,794	17	2	94.2	*C. capitata* mitochondrion, NC_000857
*Ceratitis capitata*, fresh (head/thorax)	39, 150*	0.05	2,778	25	2	96.5	*C. capitata* mitochondrion, NC_000857

1 Velvet setting: *k*-mer length, coverage cutoff and expected coverage. * Analysis was run in single-end modus.

2 The number reference-sequence alignable contigs.

3 Percentage coverage of coding sequences (CDS) is given in brackets.

4 Not shown: the read library contained extensive contamination with bacteriophage (M14428) and fungal DNA (e.g. closest related to *Aspergillus niger* rDNA AM270052).

### Alignment of fungal specimen reads

For our fungal specimens, reference nuclear scaffolds of *Agaricus bisporus* var. *bisporus* H97 v2.0 (29 scaffolds, 30.2 Mb), *Laccaria bicolor* v2.0 (55 scaffolds, 60.7 Mb), and *Pleorotus ostreatus* PC15 v2.0 (12 scaffolds, 34.3 Mb) were used, obtained from MycoCosm (http://genome.jgi.doe.gov/programs/fungi/index.jsf) and used as nuclear reference genomes. The coverage of exonic regions was estimated using the filtered set of gene models representing the best gene model for each locus, as predicted for each reference genome assembly by JGI.

For our *A. bisporus* herbarium specimen, a total of 13,330,723 (55.9% of 23,852,078) reads mapped to the *A. bisporus* reference genome assembly, and of these 10,525,133 (44.1%) mapped uniquely (Table 2). The genome coverage was 95.4% and 97.9% of the exome was covered. The average read depth was 28.7×, rising to 30× when limiting the analysis to covered regions.

Genome coverage was 71.2% (81.4% of exome) for *L. bicolor* and 78.4% (88.8% of exome) for *P. ostreatus*. The average read depths of covered regions were 29.1× and 45.6×, respectively. In general, genome and read depth coverages were evenly distributed across scaffolds of all three fungal specimens (Table S1). Scaffold 12 (281,318 nt) of *P. ostreatus*, however, was covered for only 20.6%, which is possibly due to repetitive regions.

Reads were also aligned to mitochondrial reference sequences (Table 2). Mitochondrial reference sequences for *L. bicolor* and *P. ostreatus* were assembled *de novo* (for details see de novo assembly). For all three fungal specimens we found that genome coverage was (nearly) 100%, except for inter-scaffold regions, and that average read depth was >120× (Table 2).

### Alignment of insect specimens reads

For DNA extracted from the legs of an archived specimen of *C. capitata*, a total of 24,577 (0.08% of 29,864,834) uniquely mapped reads mapped to mitochondrial reference genome NC_000857 (Table 2). These reads covered the entire mitochondrial reference genome of 15,980 nt with an average read depth of 135.4×. Comparable genome coverage (100%) and read depth (146.1×) was found for DNA extracted from the head and thorax of the (same) archived specimen of *C. capitata*.

Relatively low numbers of uniquely mapped reads mapped to *A. glabripennis* (8994; 0.02%) and *A. albopictus* (594; 0.002%) mitochondrial reference genomes (Table 2). Genome coverage for *A. glabripennis* was 90.9% (14,339 of 15,774 nt) and read depth was 37.6×. Read mapping density was low (29.4% coverage, 2.4× read depth) at the mitochondrial control region, which is likely due to the highly repetitive and AT-rich nature of this region (Quail *et al.* 2012).

The genome coverage for *A. albopictus* was 63.5% (10,582 of 16,665 nt) and the read depth was only 2.3×. *De novo* assembly revealed extensive contamination of the read library with bacteriophage (M14428) and fungal DNA (e.g. closest related to *Aspergillus niger* rDNA AM270052), and although one scaffold's (7,842 nt) best BLAST hit was with *Aedes aegypti* (AC150261), the *A. albopictus* read library was considered not suitable for further analyses. Interestingly, genome coverages and average read depths for fresh insect tissues were similar to those for archived tissues (Table 2).

### *De novo* assembly of plant specimen reads

We assessed whether genomic and chloroplast sequences could be reconstructed *de novo* from the *A. thaliana* herbarium specimen. For this, we used two different assembly strategies which were aimed at preferentially (but not exclusively) generating genomic or chloroplast contigs. The ‘best’ parameter settings for each Velvet analysis are shown in Table 3. For the assembly of genomic contigs, we used a *k*-mer length of 27 and with the minimum contig length set to 500, realizing that this *k*-mer value is substantially lower than usual in published studies. The assembly resulted in relatively short contigs, which was likely due to the inability to assemble contigs into scaffolds and the relatively low depth of read coverage of genomic sequences (∼12×; Table 2). A total of 65,388 contigs (N50 of 1,107 nt) of 500 nt or longer were produced with an assembly size of 67.1 Mb (Table 3). In order to assess the quality and similarity of the *de novo* assembly, the contigs were aligned to the TAIR10 genome. From this, 64,024 (97.9%) were alignable and covered a total of 66,082,031 nt (55.46% of the TAIR10 genome). We used the TAIR10 defined coding sequences (CDS) (ftp://ftp.arabidopsis.org/home/tair/Sequences/blast_datasets/TAIR10_blastsets/TAIR10_cds_20110103_representative_gene_model_updated) to assess the coverage of coding regions. A total of 4,431 full-length coding sequences (16.2% of 27,416) had a significant BLAT (the BLAST-Like alignment Tool) hit with the *de novo* contigs.

For assembly of chloroplast contigs, we used a k-mer value of 47 and a coverage cutoff of 50, thereby excluding low covered contigs, i.e. probably contigs of genomic and mitochondrial origin. However, not all such contigs will be relatively low coverage, for instance some mitochondrial and genomic repetitive sequences were found to be well-covered in genomic ‘shotgun’ sequencing [28] and therefore, our assembly strategy may not yield just chloroplast contigs. A total of 34 contigs with an N50 of 15,211 nt were produced. Eight contigs were alignable and covered 98.4% (152,030 of 154,478 nt) of the *A. thaliana* chloroplast reference genome. The other 26 contigs aligned to regions of mitochondrial and nuclear origin (not shown).

*De novo* assemblies of reads generated for *L. anagyroides* and *L. tulipifera* herbarium specimens were aimed at preferentially generating chloroplast contigs. For *L. anagyroides, de novo* assembly resulted in 280 contigs with an N50 of 3,614 nt. As no chloroplast reference is publically available for *L. anagyroides*, we used the chloroplast sequence of *Glycine max* (Fabaceae) as reference instead. Three contigs with a total length of 128,899 nt were alignable and covered 81.21% (123,616 of 152,218 nt) of the *G. max* chloroplast genome, which had 90.9% identity to *L. anagyroides*. The assembly of *L. tulipifera* resulted in 34 contigs (N50: 22,290 nt), of which 6 were alignable, and covering 100% (159,886 nt) of the *L. tulipifera* reference genome.

*De novo* assemblies of reads generated from plant fresh tissues resulted in comparable assembly statistics, and numbers of reference-alignable contigs and coverages as those generated for herbarium specimens (Table 3).

### *De novo* assembly of fungal herbarium reads

We assessed whether genomic and mitochondrial sequences could be reconstructed *de novo* from fungal herbarium specimens. Separate *de novo* assemblies were conducted aimed at preferentially generating either nuclear genomic or mitochondrial contigs (Table 3). For *A. bisporus*, the ‘best’ settings for the assembly of genomic contigs were found to be a *k*-mer length of 41, a coverage cutoff of 5, and an expected coverage of 50. The assembly was filtered for contigs longer than 5,000 nt. A total of 1,820 contigs (N50 of 20,217 nt) were produced with an assembly size of 27.7 Mb. These contigs were aligned to the *A .bisporus var. bisporus* H97 v2.0 reference genome. From this, 1720 (94.5%) were alignable and covered a total of 24,518,583 nt (81.10% of the *A. bisporus* reference genome). Coding sequences for the filtered set of genes models of *A. bisporus* var. *bisporus* H97 v2.0 were used for BLAT searches. From this, a total of 7,414 coding sequences (71.0% of 10,438) had a significant hit. The assembly of mitochondrial contigs resulted in 43 (N50 of 41,776 nt) contigs, of which 2 were alignable, and covered 99.9% (128,217 of 128,268 nt) of the A .bisporus mitochondrial reference genome.

For *L. bicolor*, the assembly of genomic sequences resulted in 2,559 (N50 of 19,276 nt) contigs longer than 5,000 nt, of which 2,495 were alignable, and covering 62.0% (37,617,493 nt) of the *L. bicolor* reference genome. BLAT searches with *L. bicolor* v2.0 genomic CDS regions resulted in 7,965 (34.4% of 23,130) coding sequences with a significant hit.

No mitochondrial reference genome was available for *L. bicolor* and therefore we used the mitochondrial supercontig of *Coprinopsis cinerea* okayama7#130 (http://www.broadinstitute.org/annotation/genome/coprinus_cinereus/Downloads.html), a species that also belongs to the order Agaricales [29]. We found 5 contigs with a total length of 91,035 nt and these covered only 24.5% (10,411 nt) of the *C. cinerea* mitochondrial reference genome sequence. We interpret this to be due either to low average mtDNA sequence identity of C. cinerea to *L. bicolor* (86.3%), or, alternatively, because of assembly artifacts causing high coverage contigs to represent non-mitochondrial regions predominantly.

For *P. ostreatus*, the assembly of genomic sequence resulted in 858 (N50 of 85,861 nt) contigs longer than 5,000 nt, of which 725 were alignable, and covered 78.5% (26,956,141 nt) of the *P. ostreatus* reference genome. We found 56.4% (6,959 of 12,330) of CDS regions with a significant BLAT hit (Table 3). The assembly of mitochondrial contigs resulted in 405 (N50 of 9,705 nt) contigs, of which 9 were alignable, and covering 90.18% (66,048 nt) of the *P. ostreatus* mitochondrial reference genome.

### De novo assembly of insect specimen reads

We assessed whether complete mitochondrial genome sequences could be generated from archived insect specimens. Assemblies for *C. capitata* leg and head/thorax tissue produced near identical results (Table 3). For leg tissue, 12 contigs (N50 of 5,249 nt) were generated of which one contig with a length of 15,464 nt was alignable, and covering 96.8% of the *C. capitata* mitochondrial reference genome. For head/thorax tissue two contigs were alignable, and with an identical genome coverage as for leg tissue. The mitochondrial control region (or A+T rich region) was missing from both assemblies, which is probably due to the highly repetitive nature of this region.

*De novo* assembly of *A. glabripennis* revealed contamination of the read library with bacteriophage (M14428) DNA. No contigs of mitochondrial origin could be generated, even though the library contains reads that map to the *A. glabripennis* mitochondrial reference genome (Table 2). Contamination in the read library increases the complexity of the de Bruijn graph, which probably reduced the N50-value for contigs.

*De novo* assemblies of reads generated from fresh specimens of *C. capitata* resulted in comparable assembly statistics, numbers of reference-alignable contigs and coverages as obtained with archived *C. capitata* specimens (Table 3).

### Estimates of DNA damage

We assumed that background nucleotide mis-incorporations observed in data obtained from freshly extracted DNA are due to PCR and sequencing errors that arise during the Illumina HiSeq 2000 production process, whereas added miscoding lesions in DNA extracted from collection materials are assumed to result from *post-mortem* DNA damage during specimen preparation or preservation. We used the high-coverage reads of the mitochondrial (fungi and insects) or chloroplast (plant) genomes to estimate overall substitution rate for each of six complementary nucleotide pairs among reads (Table S3).

As control ‘tissues’, Illumina HiSeq 2000 paired-end read libraries generated from DNA isolated from fresh fungal tissues were obtained from the Sequence Read Archive (SRA) at NCBI: SRR393529 (*Candida albicans* P78042), SRR427174 (*Laccaria bicolor* D101) and SRR398189 (*Serpula lacrymans*) and background mis-incorporation rates for these libraries were calculated from alignments of mitochondrial reads (Table S3).

Mismatches exhibited very low average rates (i.e. less than 0.20% per base), except for substitution class C→T/G→A in mitochondrial reads of archived insects, which was slightly elevated (max. 0.45%; Table S3). No elevated substitution rates were observed among the reads (not shown). Presumably this is a consequence of library construction procedure, which involved shearing and size-selection of random DNA fragments prior to Illumina sequencing.

One-way analyses of variance (ANOVA) were performed to test whether rates for each substitution type were higher in DNA from historical specimens than in fresh tissue. As the mis-incorporation rates calculated for *C. capitata* leg, and head/thorax historic tissue (and fresh tissue) were not independent given that the sequence libraries were derived from the same individual specimen, we performed variance analysis for the combined fungal and insect mitochondrial dataset, but excluding sample *C. capitata* head/thorax (Table S4).

No increased nucleotide misincorporation rates were detected in all historic tissues, except for A→T/T→A transversions in chloroplast DNA (*F* = 12.148; *P* = 0.025; Table S4) which had an overall very low rate (0.025%; Table S3) and therefore appear to play little or no role in damage-derived miscoding lesions in herbarium DNA.

### Genotyping of Arabidopsis and fungal and nuclear genomic sequences

For the *Arabidopsis thaliana* herbarium specimen, the resulting high confidence variants consisted of 313,690 SNPs and 49,834 indels of which 64,901 (20.7%) SNPs and 1,611 (3.2%) indels were found in CDS regions (Table S3). We found 30,165 (15.3% of 196,916) CDS regions with genetic variation. Of these, genes with highest number of SNPs and indels encode a cysteine/histidine-rich C1 domain-containing protein (NP_189287), callose synthase 11 (NP_5672780, violaxanthin de-epoxidase-related protein (NP_565520), and two pentatricopeptide repeat-containing proteins (NP_193101, NP_195043; Table S4).

Because the sequenced fungal basidiomes are dikaryotic, a state in which their cells contain two genetically distinct nuclei that are physically paired, we assessed the levels of homozygous and heterozygous SNPs. Highest numbers of heterozygous sites were found for *L. bicolor* (10,596; 1.57% of 676,973) followed by 7,095 (1.42% of 498,021) heterozygous SNPs for *P. ostreatus* (Table S5). BLASTx was used to search the non-redundant protein database for possible homologs (Table S6). Most CDS regions encode proteins with unknown functions (i.e. they encode hypothetical proteins).

## Discussion

In this study, we have demonstrated that genome-scale sequences can be generated efficiently and accurately from a wide variety of dry-preserved plant (*Arabidopsis thaliana*, *Laburnum anagyroides*, *Liriodendron tulipifera*), fungal (*Agaricus bisporus*, *Laccaria bicolor*, *Pleurotus ostreatus*) and insect (*Anoplophora glabripennis*, *Aedes albopictus*, *Ceratitis capitata*) specimens from historical collections using a standard multiplex and paired-end Illumina HiSeq 2000 sequencing procedure. We were able to produce the entire nuclear genome sequence of a 43-year-old *Arabidopsis* herbarium specimen with high and uniform sequence coverage. Moreover, we sequenced the nuclear genomes of three fungal herbarium specimens (22–82 years old) with high exome coverage, as well as the complete organellar genomes of historical specimens that were collected nearly 115 years ago, at a cost of less than 10,000 euros.

The observed rates of cytosine-to-thymine mis-incorporations which are typically elevated in ancient DNA [30], [31], but also other types of nucleotide mis-incorporations, were low and at the same level as in fresh control tissues. This supports the notion that *post-mortem* miscoding lesions are a negligible source of error in historical specimens [20], [25]. Although we used organellar DNA to assess damage, the sequence error rates in organelle and nuclear genomes have been observed not to differ [32] and, therefore, we expect that our organellar genomic results should be representative of those for nuclear genomes. Whilst the observed sequencing errors are like random noise and unlikely to produce a phylogenetic signal given sufficient read depth coverage, our data could be used for high-confidence genotyping, and selection of SNP and indel-rich coding regions that will allow for genome-scale genetic diversity and phylogenetic analyses.

We observed no chimeric read pairs, as previously reported for historical DNA [25] and we were able to make full use of paired-end information. Importantly, the amount and quality of sequence data generated from historical specimens was not inferior to sequence data of fresh tissues of the same species, showing that high quality NGS libraries can be generated from nanogram quantities of historical DNA. Alternative approaches to sequencing historical DNA, for instance through targeted enrichment, have proven successful in Cronn et al. 2012 [33], Carstens et al. 2012 [34] and Lemmon et al 2012 [35]. However, we demonstrate here that these steps may be omitted whilst still obtaining high-coverage full organellar genome sequences, as was previously shown for fresh and preserved plant tissues [27], [28], [36].

Although between 16.2% and 71.0% of coding sequence regions could be assembled *de novo* in our analyses, we remain cautious about the prospects for *de novo* assembly. In general, accurate and full *de novo* assembly of eukaryotic genomes is extremely difficult because of the considerable proportion of repetitive regions and their inherent complexity [37]. More importantly, the benefits derived from sequencing paired-ends from large insert sizes (∼4–10 Kb) generally is not practicable, as long DNA fragments are only rarely recovered from historical specimens.

As natural history collections document a permanent record of the existence of individual organisms, we attempted to maintain the integrity of each specimen by sampling no more than 5% of each specimen. For a small insect specimen such as *Aedes albopictus*, however, we unfortunately had to sacrifice the entire specimen in order to meet minimal DNA input requirements for NGS library preparation. Future research should, therefore, focus on optimizing NGS library preparation protocols and the use of single molecular sequencing technologies that require very small quantities of historical DNA [38], [39].

### Implications

In this study we show that using a standard multiplex and paired-end Illumina sequencing approach genome-scale sequence data can be generated reliably from a wide variety of dry-preserved plant, fungal and insect specimens from historical collections. We believe that our result is significant for the following reasons: i) material otherwise not available, such as rare or extinct species, or costly to obtain is now in reach for comparative genomic analyses without fully destroying the original specimen (as so far was often needed); and ii) availability of previously inaccessible genetic information from old type specimens that are crucial for resolving taxonomic uncertainties and for providing DNA barcodes for various applications (e.g. ecological studies, conservation, control of agricultural pests and pathogens); iii) accuracy of nuclear genome based phylogenetics, especially at the Angiosperm species level, is expected to be greatly enhanced as resolution will increase and organelle-transmission related artefacts (a problem with commonly-used phylogenetic markers) at the species-level can be avoided, and iv) including historical samples in demographic reconstructions will significantly increase accuracy of, for instance, estimating past effective population size.

Cost-effective sequencing of historical specimens that lack a reference genome should be possible when a nuclear genome of a closely related species is used as reference [24]. Given the continuously dropping prices for genome sequencing, the number of suitable reference genomes is expected to rise dramatically in the near future. The *A. thaliana* (157 Mb) and fungal genomes (30.2–60.7 Mb) sequenced in this study were quite small and contain relatively few repetitive regions. Therefore, even though our results will likely be directly applicable to herbarium genome assembly in genera such as *Zea* (maize) (2,500 Mb), *Triticum* (wheat) (15,000 Mb) and even *Lilium* (120,000 Mb), such projects will come with substantial costs that are currently beyond the budgets of most genome-scale evolutionary and population genomic projects. The use of new technologies such as cross-species capture hybridization and NGS sequencing of targeted loci may help to reduce genome complexity and sequencing costs [23], and would enable a tremendous increase in the quantity of comparative genomic data using historical specimens. Despite these limitations this study shows that the prospects for using historical plant, fungal and insect specimen-derived genomic data are very promising.

## Materials and Methods

### Taxon sampling

We sampled historical collection material from plants, fungi and insects for which substantial genomic information is available, including complete organellar and nuclear genome sequences, available through GenBank and/or the Genomic Encyclopedia of Fungi of the Joint Genome institute (JGI) [40] and hence allowing them to serve as controls for the efficacy of our sequening and (*de novo*) assembly methods. We collected both fresh and museum materials of the same individuals of plants (*Arabidopsis thaliana, Laburnum anagyroides, Liriodendron tulipifera*) and insects (*Ceratitis capitata, Anoplophora glabripennis*) in order to allow additional controls for (re)sequencability of reference genomes using the Illumina HiSeq2000 platform. Furthermore, comparing fresh and herbarium tissues enabled comparative statistical analyses into the spectrum of nucleotide substitutions and miscoding lesions that may result from DNA post-mortem damage.

Plant and fungal herbarium specimens were obtained from the collections of the National Herbarium of the Netherlands in Leiden (L) and Wageningen (WAG). Fresh and herbarium plant material of *Liriodendron tulipifera* L. (Magnoliaceae) and *Laburnum anagyriodes* Medik. (Fabaceae) were selected from the same individuals as previously described [20]. For *Arabidopsis thaliana* (Brassicaceae) fresh material, we selected ecotype Columbia-0, as well as a 43-year old specimen of *A. thaliana* that was collected in Beltsville, Maryland, USA by P.M. Mazzeo (Table 1; Table S2). The oldest plant herbarium material used was the specimen of *L. tulipifera* dating from 1897. Basidiomycete fungal herbarium specimens of *Agaricus bisporus* (Agaricaceae), *Pleurotus ostreatus* (Pleurotaceae) and *Laccaria bicolor* (Tricholomataceae) were selected, and, after visual examination, portions of the basidiome that lacked insect-derived damage were sampled.

Archived insect specimens were obtained from the insect reference collection of the Dutch National Plant Protection Organization (NPPO). We selected pinned specimens of the Asian tiger mosquito *Aedes albopictus* (Skuse; Diptera; Culicoidea), the Asian longhorned beetle *Anoplophora glabripennis* (Motschulsky; Coleoptera; Cerambycidae), and the Mediterranean fruit fly *Ceratitis capitata* (Wiedemann; Diptera; Tephritidae), all of which are listed as important (quarantine) pests, and had been collected and stored between 1992 and 1999 (Table 1; Table S2). We used DNA extracted from fresh material of *A. glabripennis* and *C. capitata* that had been stored in the DNA bank of NPPO since December 2010. Plant and fungal herbarium specimens were assumed to be oven-dried (60–70°C), whereas the archived insect specimens were air-dried and preserved on pins. All necessary permissions for the described plant, fungal and insect specimen sampling were obtained from the respective curators, Jan Wieringa (Wag), József Geml (L), and Bart van de Vossenberg (NPPO).

### DNA extraction and Illumina sequencing

Standard precautions to minimize contamination were employed throughout, such as using dedicated pipettes with filter tips; bleaching of forceps/pestles; and sample accessioning, DNA extractions and processing of samples for Illumina sequencing were performed in separate laboratories. Plant, fungal and insect DNAs were extracted in different laboratories.

For plants, total genomic DNA was extracted from 50 mg of leaf herbarium material or an equivalent amount of fresh leaf tissue using a modified cetyltrimethylammonium bromide method [20]. For *A. thaliana* herbarium tissue, however, 5 mg was used instead, as otherwise the entire specimen would have had to have been sacrificed, which was not allowed by the sampling protocol. DNA was eluted in 150–300 µl of pre-heated elution buffer (Qiagen).

For fungi, total genomic DNA was extracted from 30–64 mg of dried herbarium material using the Jetquick DNA purification kit (Genomed). In short, the lysis buffer was replaced and contained 1% SDS, 10 mM Tris pH 8.0, 5 mM NaCl, 50 mM molecular biological grade DTT, 10 mM EDTA and 2.5 mM PTB (N-Phenacylthiazoliumbromide) supplemented with 100 μg/ml proteinase K, based on a modification from Erickson *et al.* [41]. DNA was eluted 75–90 µl of Milli-Q water. Plant and fungal DNA extractions were visualized on 1% w/v agarose gels containing ethidium bromide, and the quantity was measured using a NanoDrop 1000 spectrophotometer (Thermo Scientific).

Insect tissues were ground with a micro-pestle in microcentrifuge tubes and DNA was extracted using High Pure PCR Template Preparation Kit (Roche) following the protocol for mammalian tissue with a final elution in 100 µl elution buffer.

Indexed Illumina library preparation and sequencing was performed at the Leiden Genome Technology Centre, Leiden University Medical Center (LGTC). DNA extracts were sheared to a 100–800 bp range using a Covaris S-series sonicator. Setting for the Covaris differed between samples according to the degree of DNA degradation. The most degraded samples were not subjected to further shearing. Barcoding adapters for multiplexing were ligated to the genomic fragments using the Paired-End DNA Sample Preparation Kit PE-102-1002 (Illumina Inc.) according to the manufacturer's protocol. We size-selected samples for ∼300 bp and enriched these fragments using 12 PCR cycles. Enriched products were run against a size standard on a 2% low-melt agarose gel at 120 V for 1 h. Complete bands were extracted from the gel, and purified with a QIAquick Gel Extraction Kit (Qiagen) according to standard protocol. Concentration and size profiles were determined on a Bioanalyzer 2100 using a High Sensitivity DNA chip. Sequencing was performed on an Illumina HiSeq 2000 Sequencing System (Illumina Inc.) using the HiSeq Paired-End Cluster Generation Kit (PE-401-1001) and HiSeq Sequencing Kit (FC-401-1001). Images were processed using Pipeline v1.9. All samples were run on two Illumina lanes (eight samples per lane) with generating paired-end 100-bp reads.

### Raw read filtering and alignment

Reads were quality-trimmed as follows; First, plots of the per base sequence quality and the per base sequence content were generated using FASTX-Toolkit 0.0.13 (http://hannonlab.cshl.edu/fastx_toolkit/index.html). Then, quality plots were visually inspected and raw sequence reads end-trimmed to a minimum 1^st^ quartile quality score of 28. Also, the first 3 or 8 nucleotides were removed from all raw sequence reads, as these positions typically had aberrant GC contents. As this phenomenon was observed in fresh as well as historic material, we feel this trimming-step will not have influenced downstream conclusions. Paired-end alignments of trimmed-reads to organellar reference genomes were performed using Bowtie 0.12.6 in the ‘–v 3’ alignment mode and with ‘tryhard’ in effect [42]. Paired-end alignments to nuclear reference genomes were performed using Bowtie 2.0.0-beta5 with ‘very-sensitive’ in effect. The SAM/BAM-alignment files were processed by filtering for reliable alignments and removing PCR duplicates using SAMtools 0.1.18 [43]. Duplicates are defined here as reads that map with identical external coordinates, and only reads with highest mapping quality were kept for further analysis. Actual numbers of mapped reads were calculated using BAMtools (https://github.com/pezmaster31/bamtools). BEDtools 2.16.2 [44] was used to calculate coverage and average read depths of the final Bowtie alignments. Quality-trimmed reads were deposited in DDBJ/EMBL/GenBank under study accessions ERP001797 to ERP001808 (Table 1).

### *De novo* assembly

*De novo* assemblies of the quality-trimmed reads were performed using Velvet 1.2.06 [45]. Since organellar and genomic sequences were expected to occur with non-uniform read coverages, we performed initial ‘parameter scans’, in which we tested a small range of *k*-mer lengths, coverage cutoffs, and expected coverages, allowing control of the output of the de Bruijn graph-based assembly (not shown). For each assembly, we estimated the output quality using the median length-weighted contig length (N50), total number of contigs and largest contig size. Final assemblies were performed in paired-end modus and with minimum contig length set to 1000 (unless otherwise specified) on a workstation with 12 CPUs (dual Intel Xeon E5645) and 64GBytes shared memory. Each analysis took ∼5–60 min to complete. Some analyses were run in single-end modus due to the limitations of our hardware. The number of alignable contigs and percentage genome coverage relative to a reference sequence were calculated using DNAdiff with default settings as implemented in MUMmer 3.07 [46]. To assess the coverage of exonic regions provided by the *de novo* assemblies, coding sequences (CDS) of reference genomes were aligned to the contigs using BLAT [47]. Hits were filtered for best matches, and a hit was considered significant if its minimal CDS coverage was 80% and with 90% or more identities.

### Rates of nucleotide mis-incorporation

The mapDamage package 0.03.3 [48] was used to compute nucleotide mis-incorporation rates along the reads (filtered for PCR duplicates) mapped onto mitochondrial (fungi and insect) or chloroplast (plant) reference genome.

Because library preparation for Illumina sequencing was performed using PCR, the actual strand of origin of potential miscoding lesions cannot be identified. Therefore, the data were summarized into scores for six complementary pairs of nucleotide substitution [49]. Average mis-incorporation rates were calculated by dividing the observed substitution counts by the A+T (or G+C) nucleotide counts in the reference genome alignment [48]. For example, the AG/TC misincorporation rate was calculated as: Observed AG/TC substitutions/A+T nucleotide count. One-way analyses of variance (ANOVA) were performed to identify if the six substitution types occurred at elevated rates in historical DNA compared to fresh control DNA.

### Genotyping of genomic sequences

SAMtools was used for calling variants in read alignment data compared to the *Arabidopsis* reference genome TAIR10 (Chr 1–5), and the JGI fungal reference genomes *A. bisporus* var. *bisporus* H97 v2.0, *L. bicolor* v2.0 and *P. ostreatus* PC15 v2.0. We used the sorted BAM files that were generated using Bowtie2, containing the reliable alignments with duplicates removed. Pileup was performed using default parameters of the ‘mpileup’ command and disabling Base Alignment Quality (BAQ) computations. Subsequently, BCFtools 0.1.17-dev was used to call SNPs and indels at each site using default settings. From these raw calls, a set of high confidence variants was created by initial filtering using ‘vcfutils.pl’, which was set to filter for read depths between 10 and 50. Additionally, variant calls were filtered out using a quality threshold of 50 for indels and 20 for SNPs. In this way heterozygous and homozygous sites were distinguished from mapping errors, sequencing errors and structural variants. BEDTools was used to calculate the number of SNPs and indels per CDS, and to identify coding sequences with 100% read coverage that display highest levels of intraspecific genetic variation.

## Supporting Information

Figure S1 **Integrity of herbarium DNA.** Top, DNA extracts from *L. anagyroides* fresh (A) and herbarium (B), *A. thaliana* herbarium (C) and fresh (D), *L. tulipifera* herbarium (E) and fresh (F), and 1 kb Plus DNA Ladder (Invitrogen), after electrophoresis on 0.8% agarose gels. Bottom, herbarium DNA extracts of *A. bisporus* (G), *P. ostreatus* (H) and *L. bicolor* (I), and HyLadder 10 kb (Denville Scientific Inc.).
(DOCX)

Table S1 **Percentage, mean and maximum read coverage over all nucleotide positions for chromosomes, scaffolds or linkage groups** (**LG**)**.**
(DOCX)

Table S2 **Specimen information.**
(DOCX)

Table S3 **Average nucleotide mis-incorporation rates** (**substitutions per base**) **observed in fresh and collection DNA.**
(DOCX)

Table S4 **ANOVA on nucleotide mis-incorporation rates for each of the six nucleotide substitution types.**
(DOCX)

Table S5 **Genotyping and total number of SNPs and indels in coding sequences** (**CDS**)**.**
(DOCX)

Table S6 **Best BLASTx hits of CDS regions with high number of SNPs or indels.**
(DOCX)
