# Supplementary material for: Genomic Treasure Troves: Complete Genome Sequencing of Herbarium and Insect Museum Specimens
Source: PLoS One. 2013 Jul 29;8(7):e69189. doi: 10.1371/journal.pone.0069189 (PMC3726723; doi:10.1371/journal.pone.0069189)
Supplement: Figure S1 — Integrity of herbarium DNA. Top, DNA extracts from L. anagyroides fresh (A) and herbarium (B), A. thaliana herbarium (C) and fresh (D), L. tulipifera herbarium (E) and fresh (F), and 1 kb Plus DNA Ladder (Invitrogen), after electrophoresis on 0.8% agarose gels. Bottom, herbarium DNA extracts of A. bisporus (G), P. ostreatus (H) and L. bicolor (I), and HyLadder 10 kb (Denville Scientific Inc.). (DOCX) [file pone.0069189.s001.docx]

**Figure S1 Integrity of herbarium DNA.**





Top, DNA extracts from *L. anagyroides* fresh (A) and herbarium (B), *A. thaliana* herbarium (C) and fresh (D), *L. tulipifera* herbarium (E) and fresh (F), and 1 kb Plus DNA Ladder (Invitrogen), after electrophoresis on 0.8% agarose gels. Bottom, herbarium DNA extracts of *A. bisporus* (G), *P. ostreatus* (H) and *L. bicolor* (I), and HyLadder 10 kb (Denville Scientific Inc.).
