# Supplementary material for: Genomic Treasure Troves: Complete Genome Sequencing of Herbarium and Insect Museum Specimens
Source: PLoS One. 2013 Jul 29;8(7):e69189. doi: 10.1371/journal.pone.0069189 (PMC3726723; doi:10.1371/journal.pone.0069189)
Supplement: Table S1 — Percentage, mean and maximum read coverage over all nucleotide positions for chromosomes, scaffolds or linkage groups (LG). (DOCX) [file pone.0069189.s002.docx]

**Table S1: Percentage, mean and maximum read coverage over all nucleotide positions for chromosomes, scaffolds or linkage groups (LG).**

| **Species** | **Chromosome** | **% read coverage** | **# mapped positions** | **# mapped positions (non-zero)** | **mean read coverage** | **Std mean read coverage** | **Max. read**  **coverage** |
| --- | --- | --- | --- | --- | --- | --- | --- |
| *Arabidopsis thaliana*, herbarium | Chr. 1 to 5 | 94.01 | 119146348 | 112003809 | 12.06 | 8.62 | 179 |
|  | Chr. 1 | 94.02 | 30427671 | 28607976 | 11.75 | 6.60 | 177 |
|  | Chr. 2 | 94.26 | 19698289 | 18566848 | 13.31 | 13.49 | 179 |
|  | Chr. 3 | 93.55 | 23459830 | 21946428 | 11.76 | 7.77 | 178 |
|  | Chr. 4 | 93.35 | 18585056 | 17349399 | 11.87 | 8.14 | 178 |
|  | Chr. 5 | 94.65 | 26975502 | 25533158 | 11.88 | 6.85 | 178 |
| *Agaricus bisporus*, herbarium*^a^* | All scaffolds | 95.62 | 30233745 | 28910121 | 28.72 | 15.40 | 181 |
|  | Scaffold_1 | 95.71 | 11617292 | 11119275 | 29.27 | 15.95 | 181 |
|  | Scaffold_2 | 98.20 | 3178543 | 3121231 | 29.53 | 14.25 | 181 |
|  | Scaffold_3 | 94.39 | 2563354 | 2419674 | 27.19 | 16.03 | 181 |
|  | Scaffold_4 | 93.71 | 2387012 | 2236988 | 27.16 | 14.17 | 140 |
|  | Scaffold_5 | 98.07 | 2368395 | 2322791 | 29.53 | 13.31 | 146 |
|  | Scaffold_6 | 95.18 | 2334609 | 2222162 | 29.37 | 14.73 | 177 |
|  | Scaffold_7 | 98.80 | 2081940 | 2057009 | 30.46 | 13.72 | 154 |
|  | Scaffold_8 | 92.21 | 1953713 | 1801513 | 26.32 | 17.29 | 179 |
|  | Scaffold_9 | 92.03 | 1748887 | 1609478 | 26.52 | 16.68 | 172 |
|  | Scaffold_10 | 93.50 | 1635119 | 1528767 | 29.70 | 20.53 | 181 |
|  | Scaffold_11 | 94.04 | 1336043 | 1256476 | 27.87 | 15.42 | 167 |
|  | Scaffold_12 | 97.78 | 1144839 | 1119478 | 30.43 | 14.42 | 130 |
|  | Scaffold_13 | 96.71 | 1061846 | 1026942 | 29.72 | 15.55 | 163 |
|  | Scaffold_14 | 95.74 | 877174 | 839830 | 29.50 | 15.51 | 142 |
|  | Scaffold_15 | 96.75 | 626669 | 606315 | 30.07 | 15.88 | 181 |
| *Pleurotus ostreatus*, herbarium | All scaffolds | 78.36 | 34343005 | 26912435 | 35.75 | 27.23 | 173 |
|  | Scaffold_1 | 84.40 | 4830258 | 4076947 | 40.31 | 25.30 | 166 |
|  | Scaffold_2 | 66.87 | 2386068 | 1595471 | 29.52 | 30.87 | 167 |
|  | Scaffold_3 | 83.13 | 4643960 | 3860418 | 39.96 | 26.89 | 167 |
|  | Scaffold_4 | 84.07 | 3603704 | 3029668 | 39.65 | 24.55 | 165 |
|  | Scaffold_5 | 86.06 | 3551996 | 3056836 | 40.39 | 25.16 | 167 |
|  | Scaffold_6 | 80.08 | 2736385 | 2191245 | 35.56 | 26.25 | 167 |
|  | Scaffold_7 | 72.52 | 3270165 | 2371569 | 31.39 | 28.77 | 167 |
|  | Scaffold_8 | 78.22 | 2708442 | 2118665 | 34.88 | 26.33 | 166 |
|  | Scaffold_9 | 71.35 | 1880400 | 1341737 | 31.08 | 28.07 | 173 |
|  | Scaffold_10 | 75.64 | 1571664 | 1188758 | 32.30 | 26.89 | 166 |
|  | Scaffold_11 | 70.27 | 2879239 | 2023249 | 29.65 | 27.88 | 167 |
|  | Scaffold_12 | 20.62 | 280724 | 57872 | 6.44 | 19.95 | 158 |
| *Laccaria bicolor*, herbarium*^b^* | All LG | 71.18 | 60668487 | 43181949 | 20.71 | 23.80 | 184 |
|  | LG_1 | 72.07 | 16917719 | 12192723 | 20.49 | 22.36 | 179 |
|  | LG_2 | 75.09 | 4752198 | 3568562 | 22.77 | 22.89 | 178 |
|  | LG_3 | 74.55 | 7958835 | 5932981 | 22.54 | 23.31 | 181 |
|  | LG_4 | 69.39 | 4336737 | 3009115 | 19.46 | 22.04 | 176 |
|  | LG_5 | 61.30 | 3262838 | 2000073 | 18.29 | 28.96 | 179 |
|  | LG_6 | 69.74 | 3662005 | 2553744 | 18.66 | 22.29 | 182 |
|  | LG_7 | 63.15 | 2600395 | 1642180 | 17.51 | 26.21 | 180 |
|  | LG_8 | 73.26 | 1948480 | 1427429 | 21.04 | 21.60 | 171 |
|  | LG_9 | 74.90 | 2164930 | 1621481 | 21.66 | 20.96 | 171 |
|  | LG_10 | 73.84 | 4309560 | 3182352 | 20.88 | 21.40 | 168 |

*^a^*calculated across all scaffolds, and separately for the 15 largest scaffolds.

*^b^*calculated across all scaffolds and linkage groups, and for individual linkage groups.
