# Supplementary material for: Genomic Treasure Troves: Complete Genome Sequencing of Herbarium and Insect Museum Specimens
Source: PLoS One. 2013 Jul 29;8(7):e69189. doi: 10.1371/journal.pone.0069189 (PMC3726723; doi:10.1371/journal.pone.0069189)
Supplement: Table S2 — Specimen information. (DOCX) [file pone.0069189.s003.docx]

**Table S2. Specimen information.**

| **Species, type of material** | **Collection number** | **Sample Origin** | **Remark** |
| --- | --- | --- | --- |
| **Plant:** |  |  |  |
| *Arabidopsis thaliana*, herbarium | P.M. Mazzeo 2667 (WAG0247594) | Beltsville, Maryland, USA |  |
| *Arabidopsis thaliana*, fresh tissue |  | NASC | Col-0 ecotype |
| *Liriodendron tulipifera*, herbarium | J.W.C. Goedhart s.n. | Leiden Hortus, NL |  |
| *Liriodendron tulipifera*, fresh tissue |  | Leiden Hortus, NL | Collected by Staats |
| *Laburnum anagyroides*, herbarium | S.J. van Oostrom s.n. | Leiden Hortus, NL |  |
| *Laburnum anagyroides*, fresh tissue |  | Leiden Hortus, NL | Collected by Staats |
| **Fungi:** |  |  |  |
| *Agaricus bisporus*, herbarium | 998173387 | Wissenkerke, Keihoogteweg NL | Collected by W. Kuys |
| *Pleurotus ostreatus*, herbarium | 937158244 | Meyendel, NL | Collected by M. Boetje-van Ruyven |
| *Laccaria bicolor*, herbarium | 990290419 | Roosendaal,, “De Moeren”, NL | Collected by E.C. Vellinga |
| **Insect:** |  |  |  |
| *Ceratitis capitata*, archived | 35 | Los Angeles, USA |  |
| *Ceratitis capitata*, fresh | 34 | Sardinia, Italy | Collected by Vincenzo Girolami |
| *Anoplophora glabripennis*, archived | s.n. | Beijing, China | Collected by Henk Stighter |
| *Anoplophora glabripennis*, fresh | 4841923 | Almere, NL | ID by Brigitta Wessels-Berk |
| *Aedes albopictus*, archived | Kweek 2003 | Crevalcore, Italy | Collected by Ernst-Jan Scholte |
