# Supplementary material for: Genomic Treasure Troves: Complete Genome Sequencing of Herbarium and Insect Museum Specimens
Source: PLoS One. 2013 Jul 29;8(7):e69189. doi: 10.1371/journal.pone.0069189 (PMC3726723; doi:10.1371/journal.pone.0069189)
Supplement: Table S3 — Average nucleotide mis-incorporation rates (substitutions per base) observed in fresh and collection DNA. (DOCX) [file pone.0069189.s004.docx]

**Table S3 Average nucleotide mis-incorporation rates (substitutions per base) observed in fresh and collection DNA.**

| **DNA sample** | **Substitution type** | | | | | |
| --- | --- | --- | --- | --- | --- | --- |
| **Plant herbarium specimen** | **(A→G/T→C)** | **(A→C/T→G)** | **(A→T/T→A)** | **(C→A/G→T)** | **(C→G/G→C)** | **(C→T/G→A)** |
| *Arabidopsis thaliana*, herbarium | 1,19E-04 | 1,06E-04 | 2,76E-04 | 7,88E-04 | 1,07E-04 | 1,02E-03 |
| *Laburnum anagyroides*, herbarium | 1,32E-04 | 1,30E-04 | 3,17E-04 | 1,81E-03 | 1,37E-04 | 1,96E-03 |
| *Liriodendron tulipifera*, herbarium | 1,09E-04 | 9,61E-05 | 1,54E-04 | 3,27E-04 | 6,48E-05 | 4,98E-04 |
| **Plant fresh tissue** |  |  |  |  |  |  |
| *Arabidopsis thaliana*, fresh tissue | 4,96E-05 | 4,46E-05 | 4,47E-05 | 1,27E-04 | 4,87E-05 | 9,03E-05 |
| *Laburnum anagyroides*, fresh tissue | 1,22E-04 | 1,21E-04 | 9,80E-05 | 1,72E-04 | 8,53E-05 | 1,98E-04 |
| *Liriodendron tulipifera*, fresh tissue | 7,14E-05 | 5,51E-05 | 6,76E-05 | 1,07E-04 | 3,45E-05 | 1,66E-04 |
| **Fungal herbarium specimen** |  |  |  |  |  |  |
| *Agaricus bisporus*, herbarium | 2,01E-04 | 1,88E-04 | 2,70E-04 | 9,73E-04 | 2,08E-04 | 5,21E-04 |
| *Pleurotus ostreatus*, herbarium | 2,70E-04 | 3,37E-04 | 3,19E-04 | 1,10E-03 | 2,74E-04 | 8,76E-04 |
| *Laccaria bicolor*, herbarium | 1,43E-04 | 1,17E-04 | 1,81E-04 | 1,16E-03 | 1,57E-04 | 6,71E-04 |
| **Fungal fresh tissue** |  |  |  |  |  |  |
| *Candida albicans* P78042, SRR393529^1^ | 4,46E-04 | 8,70E-04 | 4,97E-04 | 1,58E-03 | 2,80E-04 | 4,01E-04 |
| *Laccaria bicolor* D101, SRR427174^1^ | 1,57E-04 | 1,81E-04 | 2,55E-04 | 1,81E-03 | 2,78E-04 | 5,28E-04 |
| *Serpula lacrymans*, SRR398189^1^ | 1,62E-04 | 1,45E-04 | 1,87E-04 | 7,86E-04 | 6,97E-05 | 2,07E-04 |
| **Insect archived specimen** |  |  |  |  |  |  |
| *Aedes albopictus*, archived | nd | nd | nd | nd | nd | nd |
| *Anoplophora glabripennis*, archived | 1,39E-03 | 7,86E-04 | 1,46E-03 | 1,62E-03 | 9,84E-04 | 3,21E-03 |
| *Ceratitis capitata*, archived (leg) | 6,00E-04 | 1,41E-04 | 4,55E-04 | 2,25E-03 | 7,36E-04 | 4,49E-03 |
| *Ceratitis capitata*, archived (head/thorax) | 2,51E-04 | 8,94E-05 | 2,02E-04 | 1,04E-03 | 2,29E-04 | 2,48E-03 |
| **Insect fresh specimen** |  |  |  |  |  |  |
| *Anoplophora glabripennis*, fresh | 1,25E-03 | 4,77E-04 | 9,87E-04 | 8,69E-04 | 4,06E-04 | 2,39E-03 |
| *Ceratitis capitata*, fresh (leg) | 7,01E-04 | 1,57E-04 | 4,49E-04 | 4,28E-04 | 2,03E-04 | 2,53E-03 |
| *Ceratitis capitata*, fresh (head/thorax) | 4,11E-04 | 9,95E-05 | 2,57E-04 | 3,66E-04 | 1,33E-04 | 1,58E-03 |

*^1^*Illumina HiSeq paired-end read library downloaded from the Sequence Read Archive (SRA) at NCBI.
