# Supplementary material for: Genomic Treasure Troves: Complete Genome Sequencing of Herbarium and Insect Museum Specimens
Source: PLoS One. 2013 Jul 29;8(7):e69189. doi: 10.1371/journal.pone.0069189 (PMC3726723; doi:10.1371/journal.pone.0069189)
Supplement: Table S4 — ANOVA on nucleotide mis-incorporation rates for each of the six nucleotide substitution types. (DOCX) [file pone.0069189.s005.docx]

**Table S4 ANOVA on nucleotide mis-incorporation rates for each of the six nucleotide substitution types.**

|  |  | **Average substitution rate (Std. Deviation)** | |  |
| --- | --- | --- | --- | --- |
| **Sample type** | **Substitution type** | **Fresh tissue** | **Collection material** | **ANOVA^1^** |
| **Plant chloroplast** | (A→G/T→C) | 8,10E-05 (3,72E-05) | 1,20E-04 (1,15E-05) | *F*(1,4) = 2.991, p = .159 |
|  | (A→C/T→G) | 7,35E-05 (4,12E-05) | 1,11E-04 (1,74E-05) | *F*(1,4) = 2.049, p = .226 |
|  | (A→T/T→A) | 7,01E-05 (2,68E-05) | 2,49E-04 (8,48E-05) | ***F*(1,4) = 12.148, p = .025** |
|  | (C→A/G→T) | 1,35E-04 (3,30E-05) | 9,76E-04 (7,60E-04) | *F*(1,4) = 3.665, p = .128 |
|  | (C→G/G→C) | 5,62E-05 (2,62E-05) | 1,03E-04 (3,61E-05) | *F*(1,4) = 3.276, p = .145 |
|  | (C→T/G→A) | 1,51E-04 (5,52E-05) | 1,16E-03 (7,39E-04) | *F*(1,4) = 5.521, p = .79 |
|  |  |  |  |  |
| **Fungal mitochondrion** | (A→G/T→C) | 2,55E-04 (1,66E-04) | 2,05E-04 (6,34E-05) | *F*(1,4) = 0.242, p = .649 |
|  | (A→C/T→G) | 3,99E-04 (4,09E-04) | 2,14E-04 (1,13E-04) | *F*(1,4) = 0.570, p = .492 |
|  | (A→T/T→A) | 3,13E-04 (1,63E-04) | 2,57E-04 (7,00E-05) | *F*(1,4) = 0.303, p = .611 |
|  | (C→A/G→T) | 1,39E-03 (5,36E-04) | 1,07E-03 (9,28E-05) | *F*(1,4) = 0.995, p = .375 |
|  | (C→G/G→C) | 2,09E-04 (1,21E-04) | 2,13E-04 (5,88E-05) | *F*(1,4) = 0.002, p = .964 |
|  | (C→T/G→A) | 3,78E-04 (1,61E-04) | 6,89E-04 (1,78E-04) | *F*(1,4) = 5.001, p = .089 |
|  |  |  |  |  |
| **Mitochondrion combined,**  **Fungi + *A. glabripennis* + *C. capitata* (leg)** | (A→G/T→C) | 5,20E-04 (5,16E-04) | 5,42E-04 (4,53E-04) | *F*(1,8) = 0.005, p = .944 |
|  | (A→C/T→G) | 3,14E-04 (2,77E-04) | 3,66E-04 (3,14E-04) | *F*(1,8) = 0.078, p = .787 |
|  | (A→T/T→A) | 5,36E-04 (5,23E-04) | 4,75E-04 (3,14E-04) | *F*(1,8) = 0.051, p = .827 |
|  | (C→A/G→T) | 1,42E-03 (5,26E-04) | 1,09E-03 (5,77E-04) | *F(*1,8) = 0.872, p = .378 |
|  | (C→G/G→C) | 4,72E-04 (3,67E-04) | 2,47E-04 (1,23E-04) | *F*(1,8) = 1.678, p = .231 |
|  | (C→T/G→A) | 1,95E-03 (1,79E-03) | 1,21E-03 (1,15E-03) | *F*(1,8) = 0.608, p = .458 |

*^1^*Probability value of <.05 was considered statistically significant.
