# Supplementary material for: Genomic Treasure Troves: Complete Genome Sequencing of Herbarium and Insect Museum Specimens
Source: PLoS One. 2013 Jul 29;8(7):e69189. doi: 10.1371/journal.pone.0069189 (PMC3726723; doi:10.1371/journal.pone.0069189)
Supplement: Table S5 — Genotyping and total number of SNPs and indels in coding sequences (CDS). (DOCX) [file pone.0069189.s006.docx]

**Table S5 Genotyping and total number of SNPs and indels in coding sequences (CDS).**

| **Specimen, type of material** | **SNPs** | | | **Indels** | | **Reference genome** |
| --- | --- | --- | --- | --- | --- | --- |
|  | **Total** | **Heterozygous SNPs (%)** | **CDS** | **Total** | **CDS** |  |
| *Arabidopsis thaliana*, herbarium | 313,690 | 1,448 (0.46) | 64,901 | 49,834 | 1,611 | *A. thaliana* nuclear, GCF_000001735.3 |
| *Agaricus bisporus*, herbarium | 430,571 | 2,873 (0.67) | 183,707 | 24,161 | 1,656 | *A. bisporus* H97 v2.0 nuclear, MycoCosm |
| *Laccaria bicolor*, herbarium | 676,973 | 10,596 (1.57) | 397,800 | 26,313 | 4,408 | *L. bicolor* v2.0 nuclear, MycoCosm |
| *Pleurotus ostreatus*, herbarium | 498,021 | 7,095 (1.42) | 234,658 | 20,415 | 1,889 | *P. ostreatus* PC15 v2.0 nuclear, MycoCosm |
