# Supplementary material for: Genomic Treasure Troves: Complete Genome Sequencing of Herbarium and Insect Museum Specimens
Source: PLoS One. 2013 Jul 29;8(7):e69189. doi: 10.1371/journal.pone.0069189 (PMC3726723; doi:10.1371/journal.pone.0069189)
Supplement: Table S6 — Best BLASTx hits of CDS regions with high number of SNPs or indels. (DOCX) [file pone.0069189.s007.docx]

**Table S6 Best BLASTx hits of CDS regions with high number of SNPs or indels.**

| **Specimen, type of material** | **CDS** | **# SNPs or indels** | **CDS length (% identity)** | **Best BLASTx hit (non-redundant protein database)** |
| --- | --- | --- | --- | --- |
| *Arabidopsis thaliana*, herbarium | AT3G26550.1 (9746995 - 9749040) | 46 | 2046 (98.9) | NP_189287 [Arabidopsis thaliana] cysteine/histidine-rich C1 domain-containing protein |
|  | AT4G04970.1 (2538139 - 2542434) | 46 | 4296 (99.3) | NP_567278 [Arabidopsis thalaiana] callose synthase 11 |
|  | AT4G13650.1 (7939611 - 7942673) | 46 | 3063 (99.3) | NP_193101 [Arabidopsis thaliana] pentatricopeptide repeat-containing protein |
|  | AT2G21860.1 (9318333 - 9319607) | 47 | 1275 (97.2) | NP_565520 [Arabidopsis thaliana] violaxanthin de-epoxidase-related protein |
|  | AT4G33170.1 (15995701 - 15998673) | 48 | 2972 (99.2) | NP_195043 [Arabidopsis thaliana] pentatricopeptide repeat-containing protein |
| *Agaricus bisporus*, herbarium | Genemark.2952_g; exonNumber 1 | 93 | 2092 (97.5) | XP_001880728 [Laccaria bicolor S238N-H82] hypothetical protein |
|  | estExt_Genewise1.C_51079; exonNumber 5 | 102 | 2668 (97.9) | XP_001830599 [Coprinopsis cinerea okayama7#130] lateendosome to vacuole transport-family protein |
|  | Genemark.8911_g; exonNumber 3 | 107 | 3021 (97.5) | XP_001886815 [Laccaria bicolor S238N-H82] hypothetical protein |
|  | estExt_Genewise1Plus.C_40634; exonNumber 6 | 109 | 2800 (98.0) | EIM84127 [Stereum hirsutum FP-91666 SS1] polyketide synthase |
|  | Genemark.9379_g; proteinId 122911; exonNumber 2 | 115 | 2748 (97.7) | No significant hit |
| *Laccaria bicolor*, herbarium | e_gw1.9.1213.1; proteinId 606033; exonNumber 4 | 156 | 3428 (97.8) | XP_001889742 [Laccaria bicolor] hypothetical protein |
|  | fgenesh3_kg.LG_6_#_954_#_Locus_5360_Transcript_4/9_Confidence_0.680; exonNumber 4 | 157 | 2989 (97.3) | XP_001888692 [Laccaria bicolor] hypothetical protein |
|  | fgenesh3_kg.LG_4_#_496_#_Locus_663_Transcript_1/5_Confidence_0.733; exonNumber 3 | 169 | 3278 (96.9) | XP_001876168 [Laccaria bicolor]  RPAP1-like, C-terminal; pfam08620 |
|  | Lacbi1.Eugenev2.0000250177; exonNumber 2 | 172 | 3312 (96.5) | XP_001884455 [Laccaria bicolor] pentatricopeptide repeat domain (PPR motif) |
|  | Lacbi1.estExt_GeneWisePlus_human.C_710012; exonNumber 1 | 174 | 4023 (97.6) | XP_001889717 [Laccaria bicolor] phosphoribosylformylglycinamidine synthase |
| *Pleurotus ostreatus*, herbarium | e_gw1.02.1242.1; exonNumber 1 | 105 | 2166 (99.0) | EIM84089 [Stereum hirsutum FP-91666 SS1] ribonuclease H-like protein |
|  | e_gw1.04.44.1; exonNumber 3 | 107 | 3314 (98.8) | EGN93494 [Serpula lacrymans var. lacrymans S7.3] hypothetical protein |
|  | estExt_fgenesh1_pg.C_030495; exonNumber 7 | 113 | 2631 (98.9) | EGN93737 [Serpula lacrymans var. lacrymans S7.3] hypothetical protein |
|  | fgenesh1_pm.C_scaffold_6000226; exonNumber 11 | 113 | 2856 (98.8) | XP_002910856 [Coprinopsis cinerea okayama7#130] acetyl CoA carboxylase |
|  | gm1.5700_g; exonNumber 1 | 125 | 3195 (98.6) | XP_001877465 [Laccaria bicolor S238N-H82] hypothetical protein |
